# An Examination of Clopidogrel in the Treatment of Coronary Microvascular Disease

**DOI:** 10.7759/cureus.28406

**Published:** 2022-08-25

**Authors:** Nicholas P Iskandar, Akshay J Reddy, Allen Dang, Muhammad S Ghauri, Mildred Min, Mark Bachir, Alex Bachir, Himanshu Wagh, Nathaniel Tak, Hetal Brahmbhatt

**Affiliations:** 1 Medicine, Campbell University School of Osteopathic Medicine, Lillington, USA; 2 Medicine, California University of Science and Medicine, Colton, USA; 3 Anaesthesia, California Northstate University College of Medicine, Elk Grove, USA; 4 Neurosurgery, California University of Science and Medicine, Colton, USA; 5 Dermatology, California Northstate University College of Medicine, Elk Grove, USA; 6 Health Sciences, California Northstate University, Rancho Cordova, USA; 7 Medicine, Geisinger Commonwealth School of Medicine, Scranton, USA; 8 Medicine, California Northstate University College of Medicine, Elk Grove, USA; 9 Medicine, Midwestern University Arizona College of Osteopathic Medicine, Glendale, USA; 10 Psychiatry, Mercy General Hospital, Sacramento, USA

**Keywords:** blood clotting, cholesterol, plavix, coronary microvascular disease, dual antiplatelet therapy

## Abstract

The ability of clopidogrel (Plavix) to work in tandem with aspirin in a dual therapy strategy to boost the anti-platelet therapeutic impact and diminish platelet aggregation induced by platelet receptor inhibition is one of its many key advantages. The researchers discovered that the average reduction in risk of adverse cardiovascular events related to Plavix much outweighed any potential systemic effects. The analysis also revealed that, even though treatment results for diabetic patients with coronary microvascular disease (CMD) are poorer, the dosage and administration of clopidogrel for dual therapy are not modified to address this issue. Although it has been established that the current standard of care for microvascular disease decreases damage, more study is necessary to ensure that this standard is enhanced. It may become more usual in the future to include patient groups in trials who do not have diabetes as a criterion. Patients with diabetes often have higher low-density lipoprotein (LDL) cholesterol levels than the general population, therefore, it is possible that the research findings are flawed. To confirm or reject this assumption, further research is necessary.

## Introduction and background

Cardiovascular ailments are currently the largest cause of mortality worldwide, accounting for around 17.9 million deaths annually [1]. Therefore, it is vital that medical experts perform more studies on these concerns to prevent the deaths of numerous individuals due to cardiovascular problems. These recorded fatalities can be linked to the vast array of cardiovascular disorders that have been identified. There are four basic types of cardiovascular disease: coronary artery disease, cerebrovascular disease, peripheral artery disease, and aortic atherosclerosis [1]. This article examines coronary microvascular disease (CMD), a subset of coronary artery disease. CMD is a disorder that affects the walls and inner lining of coronary microvessels [2]. Specifically, this illness causes constriction of the tiny blood arteries around the heart. Coronary microcirculation is hindered by these constricted blood channels, resulting in unfavorable cardiovascular outcomes such as myocardial infarction (MI) [2]. Smoking, hypertension, and low high-density lipoprotein (HDL) cholesterol levels are risk factors that can lead to this disease [3-7]. Common treatments for this illness include anti-platelet medication, glycemic management, induced vasodilation, suppression of cholesterol synthesis, and primary percutaneous coronary intervention [3]. Plavix is one of the most often used main drugs (clopidogrel). Plavix is a 75 mg tablet that is typically given once a day to prevent platelets from developing dangerous clots [3]. This medication is also used to help patients deal with chest pain and improve circulation [4-8]. Plavix was prescribed around 19,448,746 times in 2019 [4]. The objective of this review is to determine how frequently physicians opt to utilize Plavix and other drugs to treat CMD patients. Additionally, this research focuses on identifying probable explanations for the widespread use of anti-platelet medication to treat CMD.

## Review

Methods

**Figure 1:**
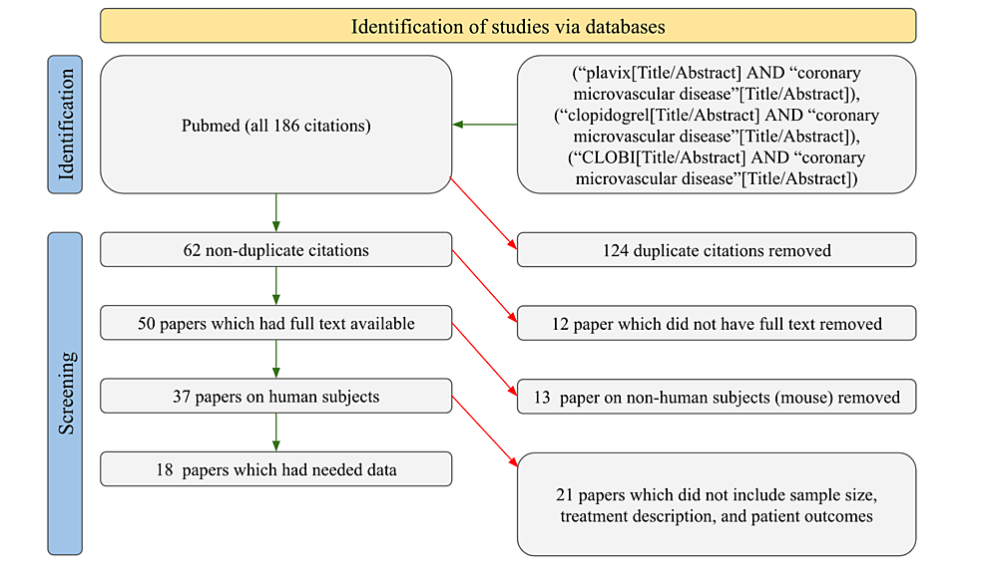
PRISMA Diagram on Study Identification PRISMA: Preferred Reporting Items for Systematic Reviews and Meta-Analyses

Searches were conducted on PubMed to find studies about the use of clopidogrel in the treatment of CMD. Exact searches were done with the keywords “Plavix and coronary microvascular disease,” “clopidogrel and coronary microvascular disease,” and “CLOBI and coronary microvascular disease.” All time frames were searched. This brought a pool of 186 studies, of which 62 were not duplicates, 50 had full text available, 37 were human studies, and only 18 had the information needed for the tables. The data collected from these 18 studies included the sample size, the type of treatment that was involved, and information regarding the patient outcomes post-treatment. Studies that did not report these results were excluded. Studies that focused on other animals such as mice were excluded. In order to remove personal bias, no studies which fit the criteria were excluded. The filtering process that was utilized by the authors for this review article can be seen more clearly in Figure 1.

Clopidogrel and CMD

CMD correlates strongly with atherosclerosis, angina, coronary spasm, endothelial dysfunction, and coronary artery disease that is not obstructive (NOCAD). More than 50% of individuals with NOCAD were also diagnosed with CMD [5]. This is crucial since NOCAD is defined as the presence of atherosclerotic plaque in blood vessels that are not expected to impede blood flow [6]. When NOCAD and CMD coexist, blood flow can be dramatically changed, leading to an increase in MI incidence. CMD is suspected to be caused by high blood cholesterol, high blood pressure, diabetes, smoking, obesity, an unhealthy diet, advancing age, and a family history of cardiovascular disease [7]. Observation of a decreased coronary flow reserve (CFR) [7] is the primary sign of the diagnosis of CMD. The capacity of tiny blood arteries to modify blood flow in response to stress is the coronary flow reserve. Therefore, coronary microvascular dysfunction has been identified as one of the specific causes in 45% of dementia cases and 25% of strokes [8]. Through a review of several research pertaining to CMD, it was shown that clopidogrel was the most commonly prescribed medicine [9-27]. Its widespread use and prescription frequency can be linked to its anti-clotting actions and superior efficacy relative to other anti-platelet medications.

In a research known as the CAPRI trial, it was shown that clopidogrel had a statistically significant benefit over aspirin in reducing the risk of adverse cardiovascular events [28]. Similar to ticagrelor, clopidogrel offers significant benefits to patients as an antiplatelet medication. In a research study, it was observed that clopidogrel and ticagrelor are equally effective against acute coronary syndrome, although clopidogrel causes a much-reduced incidence of major and minor bleeding [29]. Clopidogrel was utilized in 14 of the studies that were evaluated in our inquiry [9-11,14-17,19-27]. In these 14 trials, clopidogrel was utilized in a range of therapeutic modalities, from antiplatelet therapy to vasodilation induction. Clopidogrel does, however, produce certain severe adverse effects, including bleeding, vomiting, nosebleeds, rashes, pruritus, and thrombotic thrombocytopenic purpura [30]. Due to these serious side effects, contemporary research has begun to investigate the usage of alternative antiplatelet medications, such as ticagrelor. Ticagrelor may be able to maintain efficacy while not decreasing low shear blood viscosity and boosting adenosine generation [21]. These proposed features of ticagrelor are anticipated to increase vascular reactivity and reduce unfavorable cardiovascular events further.

Aspirin and clopidogrel in antiplatelet therapy

**Figure 2:**
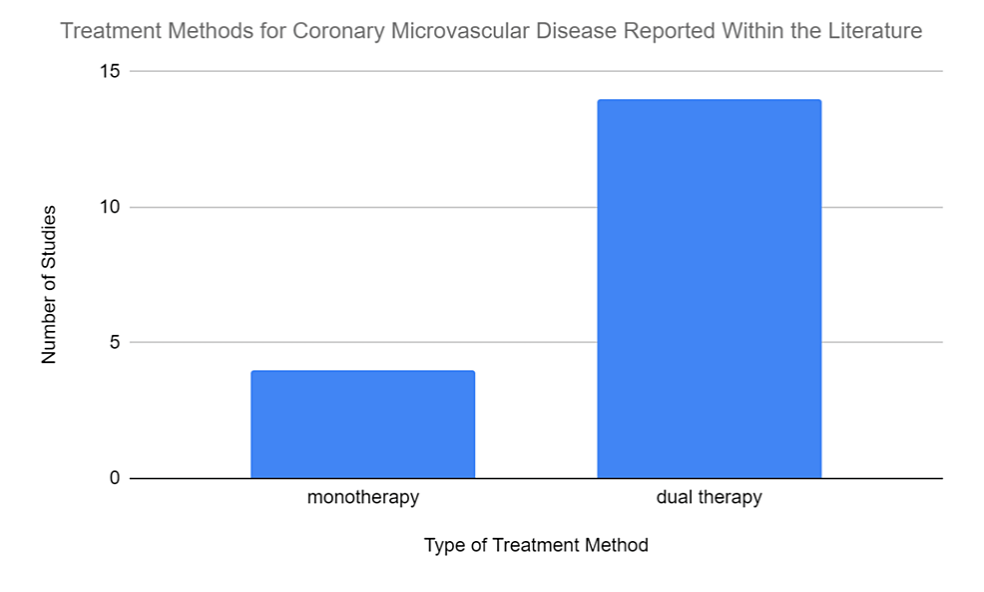
The Prevalence of Mono vs Dual Therapy

Within the review, it was discovered, through the analysis of the data in Figure 2, that 14 articles reported the usage of dual therapy to treat patients with coronary microvasculature issues [10,14-15,17-27]. This is most likely because dual antiplatelet therapy has been shown to significantly reduce the risk of recurrent stroke that patients may face if they receive solely mono antiplatelet therapy. There are several theories as to why this happens, but the most widely held theory among physicians is that having numerous antiplatelet medicines in one's system lessens the risk of clotting, which could lead to a decrease in subsequent strokes or heart attacks. The effectiveness of this combination is due to the different routes the two drugs take to affect platelets. Aspirin inhibits cyclooxygenase, preventing the production of thromboxane A(2), a vasoconstrictive agent. Whereas, Plavix works to prevent platelet activation, degranulation, and aggregation by blocking one of the three ADP receptors on the platelet surface through an irreversible binding process [31]. Therefore, Plavix is very commonly used based on studies suggesting its effectiveness in preventing platelet aggregation, its ability to be used in a dual treatment model, and its advantages in causing less major and minor bleeding. 

Six studies within the review do not report the usage of this treatment option which may suggest that while dual antiplatelet therapy is what is often used to help patients with microvascular issues, there may be instances where it is not the most conducive method of obtaining the desired outcome [9,11-13,16,18]. For instance, for patients who are allergic or intolerant to aspirin, dual therapy may not be a viable option as the treatment method commonly requires the usage of both aspirin and clopidogrel to obtain the best effect [1-3]. Another example of this would be instances where glycemic control is being used to reduce CMD issues in hypoglycemic diabetic patients [9]. Although the precise mechanism is still unknown, it has been observed that hypoglycemia in diabetic patients can result in CMD [9,32]. Because this problem is caused by blood sugar levels rather than thrombosis, or arterial clogging, patients who fall into this category are not given dual antiplatelet therapy because it does not address the underlying cause of the symptoms. Additionally, while there are multiple benefits to dual therapy, there is a significant risk that comes with it. Dual therapy has been known to cause major bleeding events for a significant number of patients who undergo the treatment [15-21]. Some physicians may be more hesitant to allow for more fragile patients to undergo dual therapy for this reason. This could also be another reason that explains why dual therapy is not a universal treatment option for patients who have coronary microvasculature issues. Additionally, if a patient has already been on dual therapy for a prolonged period of time, monotherapy might be a way for them to continue decreasing the risk of thrombosis without damaging their vascular system.

Thrombosis and atherosclerosis

The most commonly used treatment method for patients who had microvasculature issues was antiplatelet therapy. The data from Table 1 reports that 17 studies within the review reported that antiplatelet therapy was chosen by physicians as the primary method of handling coronary microvasculature issues [9-27]. The treatment modality used for clopidogrel in each study is also mentioned in Table 1. The majority of the studies that were analyzed within the review indicate that patient treatment was prescribed for at least six months [9-27]. Clogging of the arteries will usually cause a patient to experience a heart attack or a stroke. Therefore, to prevent these complications from arising in patients who have vascular problems, physicians usually prescribe treatment methods that either decrease cholesterol production or blood coagulation. Although numerous physicians tend to prescribe treatment options that decrease both of these factors for patients, medications that inhibit cholesterol synthesis, such as statins, tend to have more side effects than pharmaceuticals that hinder thrombosis [16]. This could potentially explain why anti-platelet therapy is the preferred solution to this issue. Although antiplatelet therapy is used as a means of dealing with cardiovascular issues, it is not always the universal standard of patient care due to potential complications or allergies that individuals may have. In fact, two studies within this report did not use antiplatelet therapy as the primary means of helping patients overcome their coronary microvascular issues. These publications discuss how incident rates of heart attack, stroke, and other microvasculature issues can be reduced if methods of decreasing cholesterol synthesis and glycemic control are implemented [9,18]. 

**Table 1:** Treatment Methods, Medications, and Pre-Conditions Involved With Coronary Microvascular Disease

Author (year)	Primary medication used	Usage of Aspirin in Treatment	Type of treatment	Sample size	Major Pre-condition
Aronson et al. (2014) [9]	thiazolidinedione	No	Glycemic Control	102,931	Diabetes
Cerbone et al. (2009) [10]	clopidogrel	Yes	Antiplatelet therapy	1287	Diabetes
Cerrato et al. (2017) [11]	clopidogrel	No	Antiplatelet therapy	50	Diabetes
Choi et al. (2020) [12]	ticagrelor	No	Antiplatelet therapy	61	Diabetes
D’Amario et al. (2020) [13]	ticagrelor	No	Antiplatelet therapy	100	Ischemia
Deepti et al. (2018) [14]	clopidogrel	Yes	Induced Vasodilation	1	Myocarditis
Gargiulo et al. (2016) [15]	clopidogrel	Yes	Antiplatelet therapy	1	None
Guan et al. (2021) [16]	clopidogrel	No	Antiplatelet therapy	1746	Diabetes
Khan et al. (2016) [17]	clopidogrel	Yes	Antiplatelet therapy	203	Diabetes
Kim et al. (2010) [18]	atorvastatin	Yes	Cholesterol production inhibition	171	Diabetes
Klein et al. (2004) [19]	clopidogrel	Yes	Antiplatelet therapy	1628	Diabetes
Mangiacapra et al. (2018) [20]	clopidogrel	Yes	Antiplatelet therapy	40	Diabetes
Rosenson et al. (2018) [21]	ticagrelor	Yes	Antiplatelet therapy	1581	Diabetes
Schnorbus et al. (2014) [22]	clopidogrel	Yes	Antiplatelet therapy	150	None
Sezer et al. (2007) [23]	clopidogrel	Yes	Primary percutaneous Coronary Intervention	42	Diabetes
Taylor et al. (2004) [24]	clopidogrel	Yes	Antiplatelet therapy	30	Diabetes
Undas et al. (2009) [25]	clopidogrel	Yes	Antiplatelet therapy	30	Diabetes
Weltermann et al. (2003) [26]	clopidogrel	Yes	Antiplatelet therapy	20	Diabetes
Willoughby et al. (2014) [27]	clopidogrel	Yes	Antiplatelet therapy	40	Diabetes

Patient outcomes

In order to evaluate the efficacy of the treatments that are given to patients that have CMD, it is important to determine what the long-term outcomes are for the patients post-treatment. According to the data presented in Figure 1, only 18 were available that discussed the post-treatment outcome for patients who were diagnosed with CMD. Within the review, eight of the articles discussed how post-treatment outcomes were worse for patients who had diabetes [10-12,13-15,17,19]. One of the reasons why this occurs could be because diabetes increases the risk of developing higher cholesterol levels which could lead to atherosclerosis even after treatment is administered [33-37]. Additionally, thrombosis is also more likely to occur in diabetes patients, the exact mechanism behind this is not clear, however, it has been hypothesized that changes in blood glucose and increased levels of specific lipids can trigger a platelet to start the clotting cascade within the blood vessels leading to thrombosis [38-39]. Even after treatment for CMD is given to diabetes patients with this condition, the problems associated with the disease can still resurface due to the nature of this precondition. Given that diabetic CMD patients suffer more from the symptoms, one would assume that the dosage that they are given would be highly different from that of non-diabetic CMD patients, however, according to the t-test and the p-values, presented in Table 2, that were done using the data from the literature review, it was concluded that there was no statistically significant difference from the dosage of clopidogrel that is given to diabetic and non-diabetic CMD patients. The data presented in Table 2 also reported that there was no statistical difference between the dosage given to CMD patients that had ischemia and the population of CMD patients without a history of ischemia. In order to improve patient outcomes for diabetic CMD patients, perhaps clopidogrel dosage could be a point of investigation. Although patient outcomes for diabetic CMD patients who received treatment were described as worse than non-diabetic CMD patients, the data from the literature suggests that most patients that received treatment for CMD on average had better long-term outcomes. One study, that we examined within the review, concluded that over a five-year period treatment for CMD patients reduced mortality by 5.4% [9]. Additionally, 12 studies within the review reported that post-treatment outcomes significantly reduced the risk of MI [9-18,20-21]. So although treatment options are not adjusted to patients based on their existing pre-conditions, current methods do seem to be improving the health and well-being of CMD patients.

**Table 2:** Statistical Analysis Using t-test and p-values for Clopidogrel Dosage CMD: coronary microvascular disease.

Group comparison	t-Value	p- Value
Diabetes CMD vs Non-diabetic CMD Patients	1.8729	.079471
Ischemic CMD vs Non-ischemic CMD Patients	1.7534	.1014

Existing restrictions and future applications

We were able to gather information regarding the relationship between Plavix and the treatment of CMD. Through analysis of 19 studies, we were able to gain insight into both the advantages and disadvantages of using Plavix for the treatment of CMD. Some of the main advantages of using Plavix included the reduction of platelet aggregation due to platelet receptor blocking and its ability to work in concert with aspirin in a dual treatment model to increase the antiplatelet therapeutic effect. Whereas some of its disadvantages included how it can cause an increased risk of hemorrhaging and possible skin rashes. On average, however, through the review, it was found that Plavix had a very significant effect on reducing the risk of adverse cardiovascular outcomes, far outweighing any possible side effects of the medication. While the current standard of treatment has been shown to significantly reduce harm amongst micro cardiovascular patients, further research is needed to ensure improvement of this standard. For example, several studies within the analysis included a patient population that had diabetes as a precondition before they started having coronary microvascular complications. Perhaps more studies in the future could involve patient populations that do not have diabetes as a precondition. This could be skewing the results of the studies as diabetes patients tend to have higher LDL cholesterol levels than the general population, but further research is necessary in order to verify or disprove this claim. Additionally, some of the studies did not mention how long the patients were on dual antiplatelet therapy for, and the other studies had different time-frames for this method of administration so this may also skew the data. When conducting subsequent investigations, scientists could look into how different chemicals such as ethylenediaminetetraacetic acid (EDTA) affect the health of patients who have CMD, to further our understanding of how medications can affect their health [40]. Given the recent data on the effectiveness of ticagrelor, future research should be conducted to see if on average, it can perform better than Plavix in a clinical setting. Whatever the case, further exploration into CMD is needed in order to achieve better patient outcomes.

## Conclusions

The authors complied and filtered through several studies to create a review that discussed information about the relationship between Plavix and coronary microvascular dysfunction treatment. When used to treat patients with CMD, the benefits of Plavix significantly outweigh any potential adverse effects, such as bleeding, according to the original studies that were examined in this review. Plavix offers a number of significant advantages, including the potential to work in tandem with aspirin as part of a dual therapy strategy to enhance the antiplatelet therapeutic impact and decrease platelet aggregation produced by platelet receptor blockade. However, that being said, the data from this study suggests that the treatment standard for CMD patients with pre-conditions such as diabetes was not significantly different than the treatment standards for patients without these conditions which are concerning given that diabetic CMD patients tend to have worse patient outcomes than non-diabetic CMD patients. So although the current standard for treating CMD patients has been proven to show beneficial outcomes, perhaps future investigations should attempt to verify whether adjusting this standard for diabetic patients would improve their post-treatment aftermath.
